# Chronic Fish Oil Consumption with Resistance Training Improves Grip Strength, Physical Function, and Blood Pressure in Community-Dwelling Older Adults

**DOI:** 10.3390/sports7070167

**Published:** 2019-07-09

**Authors:** Sang-Rok Lee, Edward Jo, Andy V. Khamoui

**Affiliations:** 1 Department of Kinesiology and Dance, New Mexico State University, Las Cruces, NM 88003, USA; 2 Department of Kinesiology and Health Promotion, California State Polytechnic University, Pomona, CA 91768, USA; 3 Department of Exercise Science and Health Promotion, Florida Atlantic University, Boca Raton, FL 33431, USA

**Keywords:** resistance training, fish oil, aging, muscular strength, physical function, blood pressure

## Abstract

Fish oil (FO) has received great attention for its health-enhancing properties. However, its potential synergistic effects with resistance training (RT) are not well established. The purpose of this study was to investigate the effects of FO supplementation during 12-weeks of RT on handgrip strength, physical function, and blood pressure (BP) in community-dwelling older adults. Twenty-eight healthy older adults (10 males, 18 females; 66.5 ± 5.0 years) were randomly assigned to three groups: Control (CON), resistance training (RT), resistance training with FO (RTFO). Handgrip strength, physical function [five times sit-to-stand (5T-STS), timed up and go (TUG), 6-m walk (6MW), 30-s sit-to-stand (30S-STS)], and BP were measured pre- and post-intervention. ANOVA was used with significance set at P ≤ 0.05. Handgrip strength significantly increased in RT (+5.3%) and RTFO (+9.4%) but decreased in CON (−3.9%). All physical function outcomes increased in RT and RTFO. CON exhibited significantly decreased TUG and 30S-STS with no change in 5T-STS and 6MW. BP substantially decreased only in RTFO, systolic blood pressure (−7.8 mmHg), diastolic blood pressure (−4.5 mmHg), mean arterial pressure (−5.6 mmHg), while no change was found in CON and RT. Chronic RT enhanced strength and physical function, while FO consumption combined with RT improved BP in community-dwelling older adults.

## 1. Introduction

During aging, humans inevitably undergo loss of muscle mass, which results in decline of muscular strength, metabolic rate, and respiratory function [1]. The consequences of this geriatric syndrome include increased incidence of falls [2,3,4], physical disability [5,6], and mortality [7,8]. The most severe negative outcomes may occur when they lose their functional independence, which would deteriorate the quality of their life.

As older people lose their strength and functional capacity, they tend to become more sedentary, which increases risk for metabolic (e.g., obesity, diabetes, etc.) as well as cardiovascular disorders (e.g., hypertension, heart failure, etc.). Particularly, aging-induced hypertension is considered the major contributor to coronary heart disease, stroke, and renal disorders [9]. A previous study reported that the lifetime risks for developing high blood pressure (BP) (≥140/90 mmHg) would be approximately 90% in older adults aged 55 to 65 years [10]. While sedentary lifestyle would accelerate the development of hypertension [11], physical activity has been considered the major intervention strategy to improve vascular health [12,13].

Resistance training (RT) is generally accepted as one of the most cost-effective intervention strategies to improve muscle mass and strength, and physical function, which would help older adults maintain their functional independence. A number of studies have reported improvements in mass and quality of skeletal muscle in older adults, suggesting RT as a promising intervention to combat the aging-mediated decline in physical and physiological function [14,15]. Moreover, evidence from previous research supports the beneficial effect of RT on vascular health [16,17].

Omega-3 polyunsaturated fatty acids (n-3) have attracted great attention for their health enhancing effects. Particularly, these fatty acids appear to have both anabolic and lipolytic properties [18,19]. Chronic administration of n-3 enhances muscle mass via up-regulation of protein kinase B (Akt)-mammalian target of rapamycin (mTOR)-p70 ribosomal protein s6 kinase 1 (p70s6K1) signaling in young and older healthy humans [20,21]. Further, these fatty acids combat adipocyte accumulation under a high-fat feeding condition via enhanced lipid oxidation [22]. Several studies have provided evidence that n-3 confers cardio-protective benefits, describing potent protective effects of n-3 against cardiovascular disease (CVD) [23,24,25]. In this regard, n-3 could be considered a potent therapeutic modality to combat aging-induced alterations in strength, physical function, as well as vascular health, enhancing benefits from RT in older populations. 

The primary aim of the present study was to determine the effects of daily fish oil (FO) supplementation rich in n-3 during 12-weeks of programmed RT on handgrip strength, physical function measurements associated with an ability for functional independence, and BP in community-dwelling healthy older adults. It was hypothesized that programmed RT would improve all outcome measurements, while the RT-induced benefits would be enhanced when combined with FO administration.

## 2. Materials and Methods

### 2.1. Participants

The study protocol was approved by New Mexico State University Institutional Review Board (no. 17616) for Human Subjects. Twenty-eight healthy older adults (10 males, 18 females; 66.75 ± 5.49 years) were enrolled in this study. They were considered eligible to participate in this study if they (1) were healthy without any serious cardiovascular, metabolic, neurological, or mental disorders; (2) were nonsmoker; (3) did not consume fish-oil supplements; (4) did not take anti-inflammatory drugs; (5) did not engage in strength training; and (6) did not drink excessive alcohol (no more than five drinks per week). Prior to participation, all participants were screened and admitted to the study. After the screening procedures, all participants were provided written informed consent prior to being admitted to the study.

### 2.2. Experimental Design

A longitudinal design was used to investigate changes in all outcome measurements from pre- to post-intervention. Participants visited the applied exercise physiology laboratory on the New Mexico State University campus for pre-intervention assessments including handgrip strength, physical function, and BP measurements. Upon completion of all assessments, they were randomly assigned to one of three groups: (1) control (CON, n = 8; male = 3, female = 5), (2) resistance training (RT, n = 10; male = 3, female = 7) or (3) RT with FO supplementation (RTFO, n = 10; male = 4, female = 6) for 12-weeks of intervention. After the 12-week intervention period, they visited the lab again for post-intervention assessments. All participants were instructed to maintain their regular diets and daily activity throughout the intervention period. They were also asked to avoid any strenuous exercise or physical activity for 48 h prior to their lab visit.

### 2.3. Resistance Exercise Training

The RT and RTFO groups performed programed RT twice per week for 12 weeks. We evaluated the one-repetition maximum (1 RM) of each participant in the Applied Exercise Physiology Lab before the intervention started. Subjects were asked to perform two sets of 10 repetitions or until failure (whichever came first) for 5 exercises training muscle groups in the upper and lower body (lat pull-down, seated row, biceps curl, leg press, calf rise). They performed each RT session under close supervision to ensure applying the appropriate method and to minimize the potential risk of injury. Exercise intensity was set at 50% of their 1 RM for the initial week. Then, training load was elevated to 70% of 1 RM on the second week and progressively increased (+5% weekly if they were able to complete the given work load) to promote the maximal adaptive hypertrophic response. If they failed to complete the 10 repetitions for the given workload, the same work load was given on the following session. Each exercise session was initiated with a warm-up with stretching and one set of low intensity (30% of 1 RM, 10 repetitions). Each training session was recorded in a logbook to quantify load increments.

### 2.4. Fish Oil Supplement and Diet

The FO supplement consisted of a proprietary combination of eicosapentaenoic acid (EPA; 0.7 g) and docosahexaenoic acid (DHA; 0.24 g). The supplement groups consumed 3 capsules of FO, one capsule for each meal, which provided 2.1 g EPA and 0.72 g DHA per day. The RT and CON groups received placebo capsules, which were identical in appearance (safflower oil; 3 capsules/day). This dose has been approved by the Food and Drug Administration (FDA) to effectively reduce triglyceride concentrations in those with high triglyceride levels [20]. Also, a previous study reported that whole-body protein synthesis is substantially increased with similar doses treated to rodent animals (relative to their body weight) [26], while greater doses are not effective on protein metabolism or even impair protein synthesis [27,28]. Participants consumed the supplement pills daily and returned the empty and/or remaining pills in order to ensure their compliance. The tablet counts revealed >90% compliance in each group.

### 2.5. Handgrip Strength and Physical Function Assessment

Handgrip strength measurements were performed pre- and post-intervention to evaluate strength changes in each group. To assess handgrip strength, an analogue hand dynamometer was used. Participants stood and had their elbow by their side at a right angle with a neutral wrist position. The base grip was on the first metacarpal, while four fingers were positioned on the handle. When ready, participants squeezed the dynamometer with their maximal effort for 5 s without their body moving. They performed three trials and the highest number was recorded.

Physical function was evaluated using following tests: (1) Five times sit-to-stand (5X-STS): participants performed the test on a hard, straight-backed, arm-less chair (43 cm in height) placed against the wall. With both arms crossed on the chest, they performed three trials of five times sit-to-stand at their maximal speed with a 2 min rest interval. The time was stopped with completion of fifth repetition. (2) Timed up and go (TUG): participants were instructed to sit on the chair (43 cm height) and rise, walk around a cone (2.44 m from the chair), and return back to the chair at their maximal walking speed. Three trials were given with 1 min resting interval. (3) A total of 6-m walk (6 MW): participants walked to a marked point over the 6-m line at their maximal speeds for three trials with a 30 s rest between each trial. (4) A total of 30-s sit-to-stand (30S-STS): participants were asked to repeat standing up and sitting down from a chair as many repetitions as they could for 30 s. The number of repetitions was recorded to represent their capacity.

### 2.6. Blood Pressure Measurement

Arterial blood pressure was assessed pre- and post-intervention by using an electronic blood pressure sphygmomanometer (Panasonic EW31092, Newark, NJ, USA). After 10-min rest period in a quiet environment, BP was measured twice and mean values for systolic blood pressure (SBP) and diastolic blood pressure (DBP) were recorded. If the two measurements provided values that varied greater than 2%, a third measurement was performed and the mean value of the two closest was selected for the data analysis. Mean arterial pressure (MAP) was calculated by using the following equation, MAP = [(2/3 × DBP) + (1/3 × SBP)].

### 2.7. Statistical Analysis

All data are presented as the mean ± SD. After normality assurance, 3 (experimental condition) × 2 (time) repeated measures ANOVA was used to analyze all outcome measurements. Statistical significance was set at *p* ≤ 0.05. When the significant interaction was noted, Tuckey’s post hoc testing was used to localize main or interaction effects. Cohen’s *d* effect sizes were calculated using the equation: *d* = (mean difference between pre- and post-intervention)/(pooled standard deviation). Using Cohen’s conventions, values of 0.2, 0.5, and 0.8 were considered small, medium, and large effects, respectively. Cohen’s *d* values ≥ 0.5 were interpreted as a practical/functional impact of intervention.

## 3. Results

Descriptive data of participants at baseline are provided in Table 1. There was no significant difference in age and anthropometric characteristics between groups pre-intervention.

Progression of exercise training is provided in Table 2. We observed greater than 90% adherence with similar progression of the work load in both training groups over the intervention period. Moreover, there were no significant differences in training loads between RT and RTFO throughout the intervention period.

### 3.1. Handgrip Strength and Physical Function

The data for handgrip strength and physical function are presented in Table 3.

There was a significant group × time interaction for handgrip strength. No difference was observed in handgrip strength between groups pre-intervention. Handgrip strength significantly increased in both RT (+5.3%, *p* = 0.007, *d* = 0.21) and RTFO (+9.4%, *p* < 0.001, *d* = 0.53) from baseline, while it significantly decreased in CON (−3.9%, *p* = 0.003, *d* = 0.17). 

There was a significant group × time interaction for all physical function outcomes. There was no significant difference in all physical function measurements between groups at baseline. The 5X-STS time substantially decreased in RT (−8.5%, *p* = 0.001, *d* = 0.41) and RTFO (−21%, *p* = 0.001, *d* = 1.63), but no change was found in CON. Similarly, The TUG time was significantly decreased in RT (−8.7%, *p* < 0.001, *d* = 0.77) and RTFO (−17.2%, *p* = 0.001, *d* = 1.58) from pre- to post-intervention, while CON exhibited an increase from baseline (+6.6%, *p* = 0.01, *d* = 0.21). CON demonstrated the significantly slower TUG time than RT (*p* = 0.015) and RTFO (*p* = 0.005) post-intervention. The 6MW time was significantly shorter in RT (−9.1%, *p* = 0.006, *d* = 0.73) and RTFO (−15.6%, *p* < 0.001, *d* = 2.5) than baseline, while no change was observed in CON. CON exhibited the significantly slower 6MW time than RT (*p* = 0.003) and RTFO (*p* < 0.001). The numbers of 30S-STS were substantially increased in RT (+10.6%, *p* < 0.001, *d* = 0.49) and RTFO (+21.2%, *p* < 0.001, *d* = 1.46) from baseline, but CON showed a significant decrease (−4.3%, *p* = 0.018, *d* = 0.5).

### 3.2. Blood Pressure

**Figure 1:**
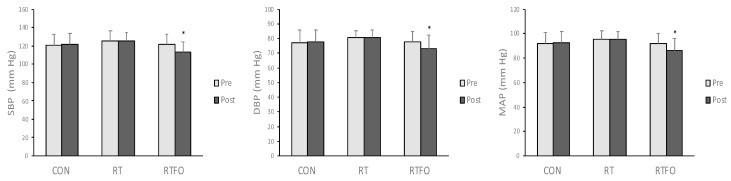
Blood pressure pre- and post-intervention. SBP = systolic blood pressure; DBP = diastolic blood pressure; MAP = mean arterial pressure. CON = control; RT = resistance training; RTFO = resistance + fish oil. Values are mean ± SD. * *p* ≤ 0.05, significantly different from pre-intervention.

The data for blood pressure are presented in Figure 1. There was a significant group × time interaction for all BP measurements (i.e., SBP, DBP, MAP). No difference was observed between groups in SBP, DBP, and MAP at baseline. RTFO exhibited a remarkable decrease in SBP (−7.8 mmHg, *p* < 0.001, *d* = 0.69) from baseline, but no notable change was found in CON and RT. Similarly, DBP greatly decreased only in RTFO (−4.5 mmHg, *p* = 0.032, *d* = 0.56) from pre- to post-intervention with no change in CON and RT. MAP substantially reduced over time only in RTFO (−5.8 mmHg, *p* = 0.002, *d* = 0.64), while no change was observed in CON and RT.

## 4. Discussion

The primary aim of the present study was to investigate the effects of FO supplementation during 12-weeks of programmed RT on grip strength, physical function, and BP in healthy community-dwelling older adults. The major findings of our study were that programmed RT improved aging-mediated declines in handgrip strength and some aspects of physical function outcomes (i.e., TUG and 30S-STS). In addition, 12-weeks of FO consumption combined with RT improved BP in older adults.

To assess muscular strength, we used the handgrip strength test, which has been widely used in epidemiology research as it is considered a strong predictor of health outcomes [29]. Leong and colleagues found that grip strength was negatively correlated with all-cause mortality in the prospective urban–rural epidemiology study in 17 countries of people with various socioeconomic statuses [30]. Their findings were supported by a current study, which described markedly lower grip strength in older adults (≥80 years) than young adults (30–39 years) [31]. We observed a significant decrease in handgrip strength in CON, which agreed with the previous evidence, while the strength decline was greatly improved by 12-weeks of programmed RT (+5.3%, *d* = 0.2) alone and combined with FO administration (+9.4%, *d* = 0.5). As grip strength is highly associated with muscle mass and age [32], our results indicate that the chronic RT-induced grip strength improvement may result from increments in muscle mass. Further, we observed the extent of the strength improvement was greater in RTFO. 

For physical function, we observed significant declines in some aspects of physical function in CON (i.e., TUG, 30S-STS), while these impairments were reversed by programmed RT. Similarly, RT exhibited great improvements in other physical function outcomes (i.e., 5T-STS, 6MW) from baseline, while RTFO produces greater treatment effects as shown in the Cohen’s *d* values. While the greater Cohen’s *d* values in RTFO appear to have practical significance of FO in physical function, these outcomes may result from the smaller standard deviation in RTFO as compared to RT as shown in the Table 3, inflating the *d* values. Future research needs to be performed to prove the potential synergistic effects of FO on RT-induced benefits in strength and physical function. These data indicate that aging-related functional decline can be combated or even reversed by regular RT and FO administration.

We also assessed changes in BP in response to 12-week of RT alone and combined with FO supplementation in older adults. Our data describe that 12-weeks of FO consumption with RT significantly decreased in BP, but no change was found in both CON and RT. While the anti-hypertensive effects of aerobic exercise in elderly have been well documented, beneficial effects of RT on BP improvements remain to be established. Delmonico et al. reported that 23-weeks of RT markedly decreased SBP and DBP in normotensive elderly [33]. Their findings agreed with those of Martel and colleagues who also found a significant decrement in both SBP and DBP in older adults after 24-weeks of RT [34]. However, Wood et al. reported that 12-weeks of RT did not influence BP in normotensive elderly [35], which was supported by the report of Anton et al. who also described no effects of 13-week of RT on BP in normotensive aged individuals [36]. The intervention period of our study was 12-weeks, similar to Wood et al. and Anton et al. and showed no change in BP in RT. Thus, it could be suggested that 12-weeks of RT would not be sufficient to improve BP in normotensive older adults. Future research will be needed to support this speculation.

On the other hand, we observed a significant decrease in BP when RT was combined with FO supplementation in older adults: SBP (−7.8 mmHg), DBP (−4.5 mmHg), and MAP (−5.6 mmHg). These findings are in accordance with previous evidence that reported FO supplementation markedly influenced BP in normotensive [37,38,39] or hypertensive [40,41] individuals. Clinical and epidemiologic evidence demonstrated cardiovascular-protective properties of FO. The primary alteration in the cardiovascular system during aging is the elevation in BP caused by structural and functional alterations in blood vessels. Specifically, aged individuals tend to lose vascular compliance, leading to increases in vascular resistance and SBP. This in turn produces a greater demand on cardiac muscle and would make the left ventricle larger and more rigid [42]. In addition, aging may decrease peripheral circulation due to impaired endothelial function in the vascular system. Endothelial cells, located between smooth muscle cells and blood, are responsible for vascular homeostasis as they regulate BP, arterial stiffness, and vascular permeability [43]. The impaired endothelial function may result from the decreased levels of vasodilator (i.e., nitric oxide) and the blunted response to stimuli of vasodilation, triggering the elevation of DBP as well as MAP [44]. The impaired endothelial function is closely correlated with many cardiovascular diseases [45].

It could be hypothesized that the way FO supplementation decreases BP is by improving endothelial cell function and thus arterial compliance. Administration of n-3 resulted in significant reduction in blood pressure accompanied with notable decrease in systemic vascular resistance by arterial vasodilation in hypertensive patients with cardiac transplantation [46]. The ways n-3 improves endothelial function include increased synthesis of vasodilators (e.g., nitric oxide) and improved vascular sensitivity to the vasodilators, lowering molecular concentration in blood vessels that is associated with an increase in arterial stiffness (e.g., thromboxane A2 or cyclic endoperoxides), and improved lipid profiles (e.g., decreased density lipoprotein cholesterol) [47]. A number of intervention studies supported the anti-obesity and anti-hypertensive effects of FO. Shen et al. found a significant decrease in low density lipoprotein-cholesterol, total cholesterol, with a great increase in high density lipoprotein-cholesterol, accompanied with a remarkable decrease in DBP after 12-weeks of n-3 administration in older adults. In addition, animal research claimed that the anti-obesity effects of n-3 would be dose dependent as increased n-3 consumption augmented the rate of body fat loss in rodents fed high-fat diet [19,48]. Taken together, the BP improvements with FO may result from increased arterial compliance with improved endothelial function in older adults. Future research will be needed to prove the suggested speculation. 

While the present study provided meaningful findings, we note that it has several limitations. First, we did not conduct biochemical and molecular analyses, which could nicely support the proposed speculations. Secondly, we could not completely control diet and physical activity levels of participants, which could result in some variations in the collected data.

## 5. Conclusions

In conclusion, 12-weeks of programmed RT combated age-related alterations in strength and physical function associated with activities of daily living, while fish oil administration combined with programmed RT improved BP in older adults. These outcomes suggest chronic RT and FO treatment as a therapeutic intervention for improving the muscular and vascular function, respectively, of older adults. Overall, our findings would provide meaningful implications for future clinical research to develop effective intervention programs for enhancing functional independence as well as cardiovascular health in older populations.

## Figures and Tables

**Table 1:** Descriptive characteristics of subjects at baseline.

Variable	CON (n = 8)	RT (n = 10)	RTFO (n = 10)
Age (years)	66.5 ± 5.0	66.6 ± 7.3	67.1 ± 4.4
Height (cm)	167.2 ± 10.24	167.9 ± 5.7	171.6 ± 9.3
Weight (kg)	68.9 ± 15.8	66.5 ± 11.5	70.8 ± 13.5
Body mass index (kg/m^2^)	24.3 ± 3.4	23.5 ± 3.6	24.0 ± 3.2

CON = control; RT = resistance training; RTFO = resistance training + fish oil. Values are mean ± SD.

**Table 2:** Progression of exercise training throughout the intervention period.

Exercise	Groups	Training Volume	Weeks 1–2	Weeks 3–4	Weeks 5–6	Weeks 7–8	Weeks 9–10	Weeks 11–12
Lat Pull-down	RT	Weight (kg)	34.1 ± 13.2	39.9 ± 17.4	44.8 ± 19.7	48.5 ± 22.2	51.7 ± 24.3	54.0 ± 25.2
		Reps	39.4 ± 2.1	38.8 ± 3.8	36.9 ± 6.0	38.2 ± 2.5	34.3 ± 7.1	35.7 ± 6.8
	RTFO	Weight (kg)	28.9 ± 10.5	36.9 ± 13.6	41.3 ± 15.7	45.3 ± 17.7	47.8 ± 17.9	50.1 ± 17.5
		Reps	40.0 ± 0.0	37.8 ± 3.5	36.4 ± 4.3	35.1 ± 7.1	33.4 ± 9.0	31.6 ± 8.9
Seated Row	RT	Weight (kg)	29.0 ± 11.4	37.0 ± 15.0	41.8 ± 16.9	44.1 ± 20.7	47.9 ± 23.9	52.3 ± 22.2
		Reps	40.0 ± 0.0	40.0 ± 0.0	40.0 ± 0.0	37.6 ± 6.2	36.9 ± 6.1	39.0 ± 1.1
	RTFO	Weight (kg)	27.8 ± 12.3	34.2 ± 13.6	38.3 ± 15.6	42.7 ± 17.5	45.5 ± 17.5	48.1 ± 16.7
		Reps	40.0 ± 0.0	40.0 ± 0.0	39.8 ± 0.6	37.3 ± 5.3	39.3 ± 1.5	38.8 ± 2.4
Biceps Curl	RT	Weight (kg)	9.4 ± 2.9	12.0 ± 3.8	13.4 ± 4.4	14.4 ± 5.2	14.7 ± 5.5	15.5 ± 6.2
		Reps	39.6 ± 1.4	39.1 ± 2.8	37.7 ± 4.0	36.9 ± 4.6	38.0 ± 2.1	38.0 ± 3.6
	RTFO	Weight (kg)	9.6 ± 3.2	12.3 ± 4.2	13.9 ± 4.9	15.4 ± 5.3	16.3 ± 5.3	18.3 ± 6.2
		Reps	40.0 ± 0.0	39.7 ± 0.9	39.2 ± 1.9	37.4 ± 5.0	38.1 ± 4.6	37.7 ± 4.6
Leg Press	RT	Weight (kg)	92.7 ± 29.8	119.3 ± 38.5	134.7 ± 43.3	148.9 ± 49.2	162.1 ± 51.9	174.6 ± 56.0
		Reps	40.0 ± 0.0	40.0 ± 0.0	40.0 ± 0.0	39.7 ± 0.8	39.2 ± 1.4	39.2 ± 1.0
	RTFO	Weight (kg)	95.3 ± 26.6	124.2 ± 32.9	137.9 ± 37.5	153.2 ± 42.7	163.7 ± 42.1	168.7 ± 47.9
		Reps	40.0 ± 0.0	40.0 ± 0.0	40.0 ± 0.0	38.5 ± 4.7	40.0 ± 0.0	40.0 ± 0.0
Calf Rise	RT	Weight (kg)	33.7 ± 11.1	43.6 ± 14.5	48.7 ± 16.4	54.0 ± 18.1	58.8 ± 19.9	63.6 ± 20.8
		Reps	40.0 ± 0.0	40.0 ± 0.0	39.4 ± 2.1	39.9 ± 0.3	39.5 ± 0.8	39.4 ± 0.8
	RTFO	Weight (kg)	35.4 ± 10.5	46.3 ± 13.8	51.1 ± 16.0	56.7 ± 18.2	60.4 ± 18.5	71.1 ± 26.0
		Reps	39.9 ± 0.2	36.6 ± 9.4	40.0 ± 0.0	37.7 ± 4.9	39.5 ± 0.9	37.3 ± 6.7

RT = resistance training; RTFO = resistance training + fish oil.

**Table 3:** Handgrip strength and physical function pre- and post-intervention.

Variable	CON (n = 8)		RT (n = 10)		RTFO (n = 10)	
	Pre	Post	Pre	Post	Pre	Post
Handgrip strength (kg)	26.8 ± 6.6	25.8 ± 6.3 *	28.5 ± 6.8	30.0 ± 7.6 *	26.7 ± 4.6	29.2 ± 4.8 *
5X-STS (s)	6.7 ± 1.0	6.9 ± 1.0	7.1 ± 1.6	6.5 ± 1.3 *	7.5 ± 1.2	5.9 ± 0.7 *
TUG (s)	6.1 ± 0.6	6.5 ± 0.6 *	5.7 ± 0.7	5.2 ± 0.6 *!	5.8 ± 0.8	4.8 ± 0.4 *!
6MW (s)	3.7 ± 0.5	3.8 ± 0.4	3.3 ± 0.5	3.0 ± 0.3 *!	3.2 ± 0.2	2.7 ± 0.2 *!
30S-STS (repetition)	23.5 ± 1.9	22.5 ± 2.1 *	20.7 ± 4.9	22.9 ± 4.1 *	19.3 ± 2.9	23.4 ± 2.7 *

5X-STS = five times sit-to-stand; TUG = timed up and go; 6MW = 6-m walk; 30S-STS = 30-s sit-to stand. CON = control; RT = resistance training; RTFO = resistance training + fish oil. Values are mean ± SD. * *p* ≤ 0.05, significantly different from pre-intervention; ! *p* ≤ 0.05, significantly different from CON post-intervention.
